# Effect of Foam on Liquid Phase Mobility in Porous Media

**DOI:** 10.1038/srep43870

**Published:** 2017-03-06

**Authors:** A. A. Eftekhari, R. Farajzadeh

**Affiliations:** 1 Centre for Oil and Gas, Technical University of Denmark, Kgs. Lyngby, Denmark; 2 Delft University of Technology, The Netherlands; 3 Shell Global Solutions International, Rijswijk, The Netherlands

## Abstract

We investigate the validity of the assumption that foam in porous media reduces the mobility of gas phase only and does not impact the liquid-phase mobility. The foam is generated by simultaneous injection of nitrogen gas and a surfactant solution into sandstone cores and its strength is varied by changing surfactant type and concentration. We find, indeed, that the effect of foam on liquid-phase mobility is not pronounced and can be ignored. Our new experimental results and analyses resolve apparent discrepancies in the literature. Previously, some researchers erroneously applied relative permeability relationships measured at small to moderate capillary numbers to foam floods at large capillary number. Our results indicate that the water relative permeability in the absence of surfactant should be measured with the capillary pressure ranging up to values reached during the foam floods. This requires conducting a steady-state gas/water core flood with capillary numbers similar to that of foam floods or measuring the water relative-permeability curve using a centrifuge.

Foams are used in several subsurface applications ranging from aquifer remediation^1^ to maximizing oil extraction from hydrocarbon fields^2^. The purpose is either to block the high-permeability layers with foam and divert the fluids into the low-permeability layers or to create a viscous pressure gradient to oppose gravity override.

Foam exhibits two flow regimes depending on the gas fractional flow (i.e., foam quality) in porous media^3^. In the *high-quality* regime, the pressure gradient along the core is nearly independent of gas superficial velocity, while in the *low-quality* regime, the pressure gradient is nearly independent of liquid superficial velocity. For a constant total superficial velocity, the high-quality regime is the range of foam quality where the pressure gradient decreases with an increasing foam quality, whereas in the low-quality regime the pressure gradient increases with the increasing foam quality.

Most foam models currently in use are built on the concept of the *limiting capillary pressure (P*_c_^*^), above which foam becomes unstable^4^,^5^. A basic assumption of these models is that foam in porous media only affects the gas mobility (quotient of the gas relative permeability by its viscosity) and the liquid mobility remains unchanged. This of course simplifies the models and reduces the number of the input parameters based on the experimental observations^6^,^7^,^8^,^9^,^10^. Bernard *et al*.^10^ were the first to conclude that for a given water saturation presence of foam does not affect liquid relative permeability. Figure 2 in ref. 10 compares the water relative permeability in presence and absence of the foaming agent in a 3.5-Darcy sandpack. At 0.01 wt% and 1.0 wt% surfactant concentrations, the water relative permeability deviates from the rest of the data points. The difference becomes even more pronounced when the data points are plotted on a logarithmic scale. At the lower concentration of 0.01 wt%; however, the presence of foam does not change the water relative permeability. Similar conclusions have been reported by other researchers, although a detailed analysis of the results may lead to a different outcome^11^,^12^,^13^,^14^.

Some studies do suggest the change of water relative permeability during foam flow in porous media; although the exact relation is not clear. De Vries and Wit^15^ predicted that the water relative permeability at high-quality regime does not monotonically increase with water saturation. In the experiments of Aarra *et al*.^16^, the water relative permeability after foam injection for both N_2_ and CO_2_ foams experienced a significant reduction with an implication that foam can be used to divert liquid among layers. Rossen and Boeije^17^ closely examined the data presented by Persoff *et al*.^18^ and Ma *et al*.^19^ and concluded that the data are not consistent with the approximation that liquid relative permeability is not affected by foam properties. They emphasized that accurate measurements of liquid saturation and relative permeability are key to upscale the steady-state foam data to a surfactant-alternating-gas (SAG) flood.

There is a lack of a systematic and comprehensive study on the effect of surfactant concentration and foam properties on the liquid relative permeability in presence of foam. Therefore, it is our objective to approach this problem by conducting foam-injection experiments with different surfactant types and concentrations. A medical CT scanner is used to obtain accurate liquid-saturation profiles at steady-state condition (key parameter in estimation of the relative-permeability functions and foam parameters) during the co-injection of gas and surfactant into a Bentheimer sandstone core.

## Materials and Methods

### Material

Anionic C_14–16_ Alpha Olefin Sulfonate (AOS, Stepan) and amphoteric Capryl/Capramidopropyl Betaine (Amphosol, Stepan) are used as received. Nitrogen is supplied from a 200-bar gas cylinder with a purity of 99.98%. Isopropyl alcohol with a purity of 99.7% is used to “kill” the foam after each experiment. The critical micelle concentration (CMC) of AOS and Amphosol in demineralized water are measured to 0.08 wt% and 0.002 wt%, respectively. Surface tensions of 38.4 ± 1, 32.5 ± 1, and 33.9 ± 1 mN/m are measured for the solutions with AOS concentrations of 0.03, 0.10, an 0.50 wt%, respectively. The surface tension of the 0.50 wt% Amphosol surfactant solution is measured to be 33.4 ± 1 mN/m.

### Experimental setup

The core-flood experiments are performed in a set-up shown schematically in Fig. 2. A Bentheimer core (*L* = 17 cm, *D* = 3.8 cm, φ = 0.21, permeability = 2.41 × 10^−12^ m^2^) is drilled out of an outcrop block and dried in oven for 48 hours. The outside of the cylindrical core is confined by covering it with Araldite epoxy. The glued core is placed into a PEEK (polyether ether ketone) core holder with no space in between, which is suitable for CT-Scan analysis because of its low X-ray attenuation. The confining or overburden pressure equal to the injection pressure is applied. The injected gas-to-liquid ratio is controlled by adjusting the set-points of the pump and the mass-flow controller. Two pressure transducers measure the pressure drops in the middle and over the core length. The outlet of the core holder is connected to a back-pressure regulator to maintain a constant pressure. The experiments are conducted at *T* = 22 °C and a back pressure of 95 bar. All the measurements are digitally stored every five seconds. A medical CT-scan instrument is used to monitor the saturations. The core holder is installed vertically using a poly-methyl-methacrylate stand at the front edge of the table^8^,^20^. Each scan contains four slices, which cover a vertical 2.5 mm thick cross-section from the middle of the core. The resolution of each image is 521 × 521 pixels, with a pixel size of 0.3 × 0.3 mm.

### Procedure

The leakage-proof setup is flushed with CO_2_ at atmospheric pressure to remove air from the system and then vacuumed for 12 hours to remove the CO_2_. Next, 10 to 15 pore volumes of water are injected with increasing steps of 5 bar to a maximum pressure of 20 bar to dissolve and remove the remaining traces of CO_2_, and fully saturate the core with water. Afterwards, several pore volumes of the surfactant solution are injected to satisfy the adsorption capacity of the rock surface. Nitrogen gas and surfactant solution with different flow rates are co-injected into the core with total flow rate (gas + liquid) of 1.0 ml/min (4 ft/day Darcy velocity). Gas and liquid are coinjected until steady-state pressure drops are obtained. The core is scanned after reaching each steady state (*HU*_*foam*_).

The liquid saturation, *S*_*w*_, in the core is calculated by


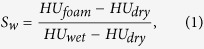


where *HU* denotes the attenuation coefficient in Hounsfield scale, and subscripts *dry* and *wet* correspond to *S*_*w*_ = 0 and *S*_*w*_ = 1 steps, respectively.

## Results and Discussion

**Figure 1:**
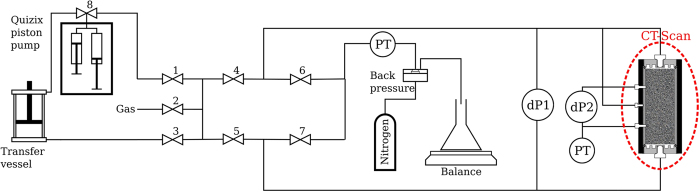
The schematic of the experimental set-up.

**Figure 2:**
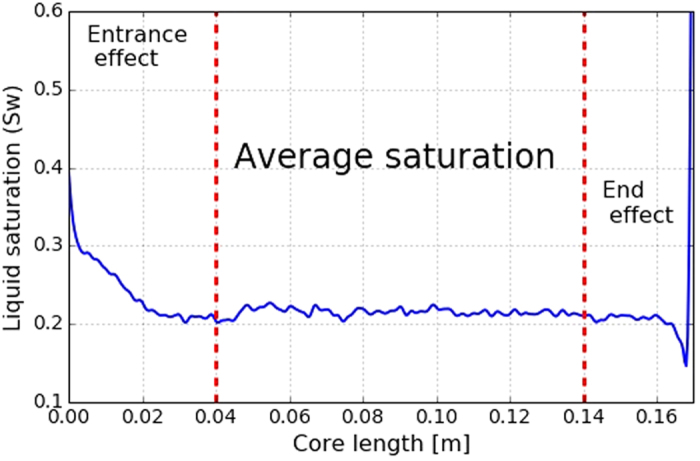
The average liquid-saturation profile in the core with marked entrance and end effects.

The measured pressure-drop data are reported in terms of the apparent viscosity of foam, *μ*_*foam*_ [Pa.s], at different qualities, *f*_*g*_ [−], which are defined by


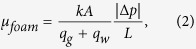



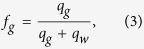


where *A* [m^2^] is the cross-sectional area of the core, *q*_*g*_ [m^3^/s] and *q*_*l*_ [m^3^/s] are the injection rates of gas and liquid, *k* [m^2^] is the absolute permeability, Δ*p* [Pa] is the pressure drop across the core length, *L* [m]. The relative permeabilities of the aqueous phase, *k*_*rw*_, and the gas phase, *k*_*rg*_, is calculated by rearranging the extended Darcy’s law and using the above definitions, i.e.,


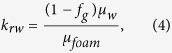



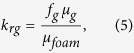


where *μ*_*g*_ [Pa.s] and *μ*_*w*_ [Pa.s] are the viscosities of the gas and liquid phases, respectively. The liquid-saturation measurements are performed for a section from the middle of the core, where the average saturation is not affected by beam-hardening, the entrance and capillary-end effects^21^,^22^, as shown in Fig. 1.

**Figure 3:**
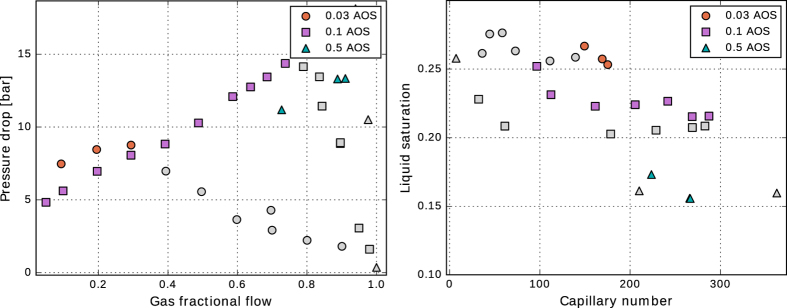
Measured pressure drops for different surfactant concentrations and the corresponding capillary numbers calculated from Eq. (6).

The gas/water relative-permeability data should be measured with large viscous forces, i.e. large capillary numbers, to remain invariant to changes in the reservoir conditions and consistent with the derivation of the relative permeability from fractional-flow theory described by Eqs 3 and 4 23. When surfactant is present in the porous medium formation of foam creates large pressure gradients leading to increase in the corresponding capillary numbers given by^24^


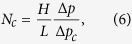


where, *H* and *L* are the diameter and the length of the core and Δ*p*_*c*_ is a characteristic difference in the capillary pressure and is taken to be 1000 Pa^23^. Using the measured pressure-drop data shown in Fig. 3a and the measured water saturations from the CT scanner for different foam qualities and surfactant concentrations, a plot of the liquid saturation vs the capillary number can be constructed, which is shown in Fig. 3b. The dark-colored points are the measurements in the low-quality regime and the gray-colored symbols are the data in the high-quality regime. It is interesting to note that for a given capillary number multiple liquid saturations are obtained. This is because the rate of coalescence of foam increases when liquid saturation decreases. This liquid saturation, known as the limiting water saturation (*S*_w_^*^), is dependent on the surfactant concentration^4^,^5^. It can be inferred from the magnitude of the capillary number that our measurements are performed at large capillary numbers and therefore the flow (specially in the low-quality regime) is viscous dominated, i.e. the calculated water relative-permeability data in this paper are the *intrinsic* relative permeabilities of the system and are not affected by the capillary-pressure heterogeneity. The calculated capillary numbers for some of the data points in the high-quality regime are small and therefore in our analysis we use the saturation data from the low-quality regime to estimate the relative-permeability functions.

### Liquid phase relative permeability

**Figure 4:**
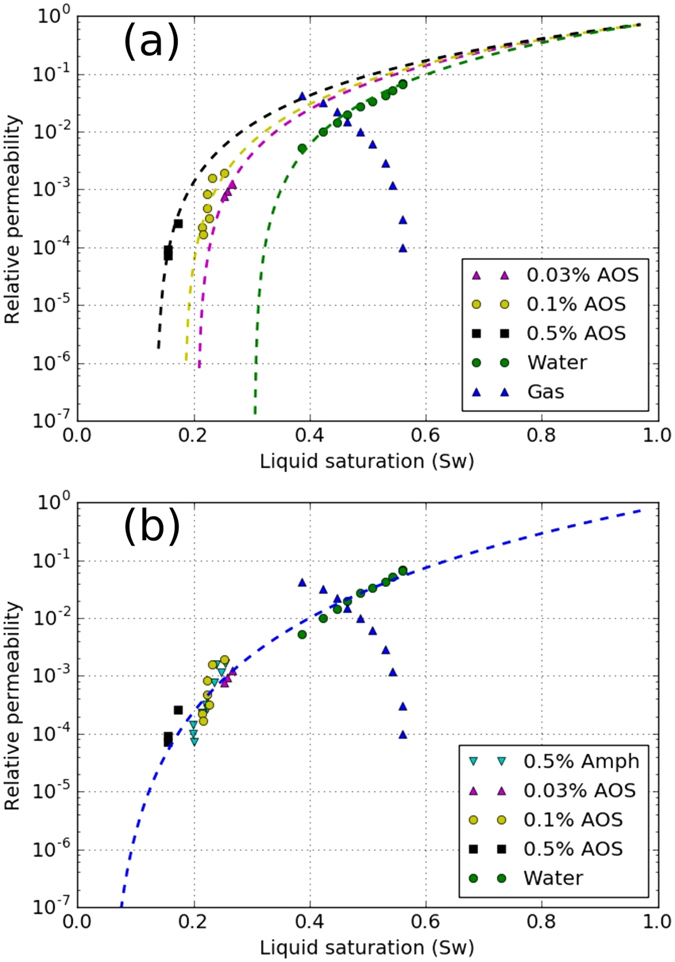
Gas and liquid permeability data in the absence of the surfactant, and liquid permeability data in the presence of AOS and Amphosol surfactants in a Bentheimer sandstone; the top figure shows four different relative-permeability curves with different values assigned for *S*_*wc*_. The bottom figure shows a single water relative-permeability curve fitted to all the data points; the parameters are shown in Table 1.

The Corey-type relative-permeability model fitted to the experimental data, shown in Table 1, is defined by


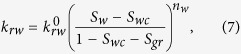



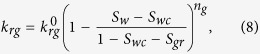


where 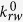 and 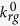 are the end-point permeabilities for the water and the gas phase, *S*_*wc*_ is the irreducible-water saturation, and *S*_*gr*_ is the residual-gas saturation. A major difficulty in the measurements of the gas/water relative-permeability data is to determine *S*_*wc*_, i.e. the water saturation at which the water relative permeability approaches zero. The reported values of *S*_*wc*_ are often impacted by the relative magnitude of gravity/capillary/viscous forces and are not the intrinsic property of the rock. Such measurements lead to an *effective k*_*rw*_ function and may be misleading when foam data are interpreted. Here, we examine two sets of *k*_*rw*_ data to investigate the effect of foam on liquid phase mobility. The first set includes the data measured using the steady-state gas/water displacement experiment at low capillary numbers (Fig. 4a). The second set of data is taken from ref. 23 measured at high capillary numbers (Fig. 4b). In our optimization scheme, we first fit the gas and water relative-permeability curves to the gas/water relative-permeability data. We examined various optimization schemes to fit *k*_*rw*_ function to the *k*_*rw*_*-S*_*w*_ data obtained from the foam experiments. For the first set of data the best fit is obtained by keeping the values of 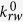, *n*_*w*_, and *S*_*gr*_ constant and only varying the irreducible water-saturation value, whereas for the second set of data a single curve is obtained for all liquid relative permeability data. These optimized values of the Corey-model parameters are used for comparing the implicit-texture foam model to the experimental data.

### Gas phase relative permeability

In the implicit-texture (IT) local-equilibrium foam model, for a two-phase flow system, the gas relative permeability in the presence of foam is given by


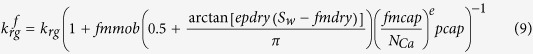


where *N*_*Ca*_ = *μ*_*foam*_*u*_*t*_/*σ*_*wg*_ is the reference capillary number, and [*fmmob, epdry, fmdry, fmcap, epcap*] are the adjustable parameters used to explain the physics of foam flow in porous media^25^. Among these parameters, *fmdry* represents the *limiting water saturation, S*_w_^*^. Below *fmdry* (above *P*_c_^*^) foam dries out and its coalescence rate increases dramatically leading to a coarse-texture foam. If foam collapse is abrupt (large *epdry*), the transition between regimes occurs at 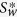. However, if foam collapse is not abrupt (smaller *epdry*), there is a range of water saturations over which foam becomes coarser in texture.

**Figure 5:**
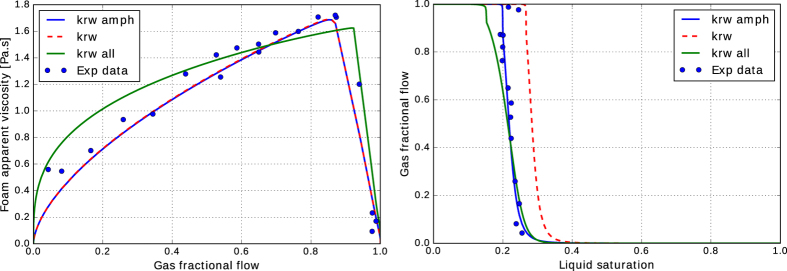
Experimental data for the measured apparent viscosity and saturations as functions of the gas fractional flow for the flow of 0.5 wt% Amphosol nitrogen foam in a Bentheimer sandstone core. The solid lines are calculated using the IT-foam model. The green line assumes that the liquid mobility remains unchanged in the presence of foam and uses a single relative-permeability curve with the parameters shown in Table 1. The blue line assumes that the presence of foam alter the value of S_wc_ in the first set of relaive-permeability data.

The liquid relative permeabilities in the presence of the surfactant, calculated by Eq. (4), are shown in Fig. 4. When the first set of the relative-permeability data is used the calculated liquid relative-permeability values in the presence of the surfactant do not coincide with the measured surfactant-free gas/water relative-permeability curve. The closest surfactant relative permeability is for 0.03 wt% AOS, i.e., the lowest surfactant concentration in our experiments. The *k*_*rw*_ - *S*_*w*_ curve shifts to the left by increasing surfactant concentration and the *apparent* irreducible-water saturation decreases from 0.25 in the original water relative-permeability curve to 0.135 for the 0.5 wt% AOS, as shown in Table 1. This may lead to the conclusion that the liquid relative-permeability parameters are functions of the surfactant concentration in the presence of foam in porous media. With this assumption the measured apparent viscosity data can be compared to the IT foam model. As an example, the data for the flow of 0.5 wt% Amphosol/nitrogen foam is shown in Fig. 5. The solid line shows the IT-foam model fitted to the data, following the procedure of ref. 5. The adjusted liquid relative permeability is employed to obtain an agreement between the data and the IT-foam model. With the assumption of an unaltered liquid relative permeability in the presence of foam, one does not need to measure the liquid saturation because it can be back-calculated from Eq. (4) using Eq. (5):


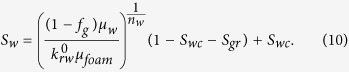


The relative permeability of the gas phase can be obtained by fitting the model to the *μ*_*foam*_ − *f*_*g*_ data. Figure 5 compares the match between the IT-foam model and the data using the modified and the surfactant-free liquid relative permeability values. In both cases, it is possible to obtain a good match with the apparent-viscosity data; nevertheless, the *f*_*g*_ *– S*_*w*_ data can only be matched with the altered liquid relative-permeability curve (solid blue curve). This confirms that without exact information about the liquid saturation in the porous medium, estimation of foam-model parameters from apparent-viscosity data can be erroneous.

The estimated foam-model parameters using the two sets of relative-permeability data are provided in Table 2. The results show that for the first set of relative-permeability data, *fmdry* is the only parameter that differs for the cores with and without the surfactant solution. The value of *fmdry* cannot decrease below the irreducible-water saturation, i.e., *fmdry* > *S*_*wc*_. Therefore, when the value of *S*_*wc*_ decreases in the relative-permeability function (Eq. 5), the value of *fmdry* is also allowed to reach lower values. Consequently, a fractional-flow curve that matches the data is obtained. The value of *fmdry* in the simulation has a significant impact on the ultimate stability and sweep of foam in porous media.

However, the surfactant-free *k*_*rw*_ data used in this approach is questionable, because the data has been measured at low capillary numbers or capillary-dominated flow without considering the capillary pressure^26^,^27^. In such an experiment, the capillary-pressure profile decreases from the value at the inflow end to a value of zero at the outflow end of the core. Most likely the reported connate water saturation of *S*_*wc*_ = 0.25 is not the water saturation at which the water relative permeability goes to zero but rather it is the water saturation at which water no longer flows because of dominance of the capillary pressure in the absence of the surfactant.

When surfactant is present in the core, injection of the gas creates foam, which in turn induces large pressure gradients and changes the force balance in favor of viscous forces. During foam-displacement experiments, the magnitude of the capillary pressure becomes almost equal to the limiting capillary pressure, and the water saturation close to the limiting water saturation or *fmdry*. Therefore, it can be argued that the relative-permeability curves in the absence of surfactant could be used only if the gas pressure gradient is approximately equal to that of the foam floods, i.e. in the limit of viscous-dominated flow or large capillary numbers. Then the capillary pressure (and distribution of the liquid) is similar to that of the foam floods and the measured liquid relative permeability will be much closer to the correct value. To assess this argument, a single liquid relative-permeability curve (with the parameters in the last row of Table 1 and shown in Fig. 4b) is used. In this approach the measured liquid saturation for each foam quality in the experiments is assigned to be the *fmdry* value in Eq. (9). The green curves in Fig. 5 show the comparison between the data and the results of the IT foam model for the experiment with 0.5 wt% Amphosol surfactant. A satisfactory (but not perfect) agreement is obtained for both apparent viscosity and fractional-flow curves. The foam-model parameters for the 0.5 wt% Amphosol surfactant case are shown in the last row of Table 2, which are different than the ones from the first approach. This implies that choosing *k*_*rw*_ data measured at large capillary numbers, i.e. the intrinsic relative permeability, leads to the conclusion that the effect of foam on the liquid phase mobility is not pronounced and can be ignored. The deviation of the calculated fractional-flow curve (green line in Fig. 5) at higher foam qualities is likely because of the difficulties in reaching steady-state point at high foam qualities.

### Foam strength

**Figure 6:**
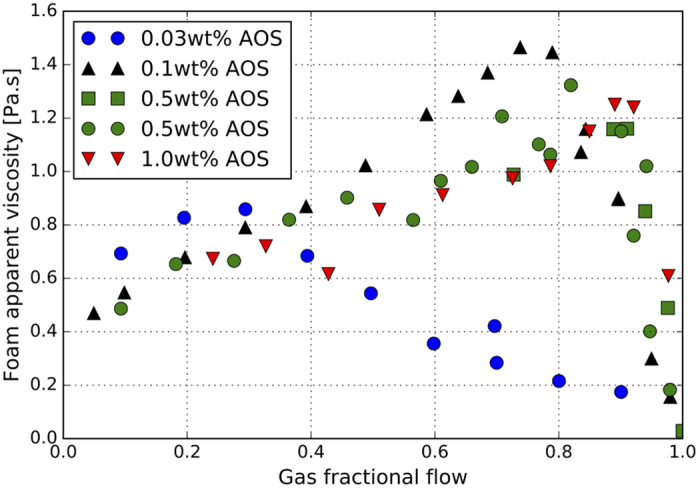
Effect of surfactant concentration on foam apparent viscosity.

Figure 6 shows the apparent viscosity of AOS/nitrogen for different foam qualities and AOS concentrations. The transition foam quality 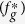, i.e., fractional flow at which *μ*_*foam*_ reaches its maximum, occurs at a larger value when surfactant concentration increases. When surfactant concentration increases from 0.03 wt% to 0.1 wt%, 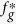 jumps from 0.25 to 0.8. However, at concentrations above CMC, i.e., 0.1 wt%, 0.5 wt%, and 1.0 wt%, the rate of increase of 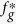 with surfactant concentration reduces considerably. At low-quality regime, the relationship between the surfactant concentration and the foam strength is not obvious for concentrations above the CMC. However, foam becomes stronger when concentration drops below the CMC. The maximum apparent viscosity of foam first increases from 0.9 Pa.s (at 0.03 wt% AOS) to 1.4 Pa.s (at 0.1 wt% AOS). For concentration above 0.1 wt%, the maximum apparent viscosity slightly decreases with increasing surfactant concentration. This is likely because at high surfactant concentrations when the concentration of micelles in the surfactant solution is high, some micelles are captured in the film during the thinning process^8^,^19^,^20^.

Foam stability in porous media is determined by the magnitude of *P*_c_^*^, which depends on surfactant type and concentration, among other parameters^4^,^28^. It can be inferred from the experimental data at high-quality regime that *P*_c_^*^ increases with increasing surfactant concentration because of the lower water saturations and results in stronger and more stable foam. In the low-quality regime foam rheology is more complex and is a function of many factors including gas trapping and the resistance to individual lamellae as they flow. The relationship between foam strength at low-quality regime and surfactant concentration requires more investigation.

## Conclusions

This study confirms that the effect of foam on the mobility of the aqueous phase in porous media is not pronounced and can be ignored. The contradictory results in literature are attributed to the erroneous choice of the surfactant-free relative-permeability curves. For the systems used in this study, when the steady-state gas/liquid relative permeability data (measured at low capillary numbers) is used, the aqueous phase relative permeability in the presence of foam appears to depend on the surfactant concentration; in particular, the *apparent* irreducible water saturation (*S*_*wc*_) decreases by increasing the surfactant concentration. Nevertheless, when the relative-permeability is measured at large capillary numbers, the effect of foam on the mobility of the liquids phase becomes insignificant. This indicates that the water relative permeability in the absence of surfactant should be measured with the capillary pressure ranging up to values reached during the foam floods. This will require conducting a steady-state gas/water core flood with capillary numbers similar to that of foam floods or measuring the water relative permeability curve using a centrifuge.

## Additional Information

**How to cite this article**: Eftekhari, A. A. and Farajzadeh, R. Effect of Foam on Liquid Phase Mobility in Porous Media. *Sci. Rep.* **7**, 43870; doi: 10.1038/srep43870 (2017).

**Publisher's note:** Springer Nature remains neutral with regard to jurisdictional claims in published maps and institutional affiliations.

## Figures and Tables

**Table 1:** Optimized parameters of the Corey relative-permeability model for air-water flow in the Bentheimer sandstone; the parameters for the gas phase relative permeability in absence of surfactant are 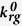 = 0.587; *n*_*g*_ = 0.938; *S*_*gr*_ = 0.03.

Parameters	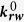	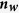	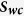
No surfactant	0.713	2.460	0.25
0.03% AOS	0.713	2.460	0.200
0.1% AOS	0.713	2.460	0.184
0.5% AOS	0.713	2.460	0.135
0.5% Amph.	0.713	2.460	0.181
All data	0.720	4.423	0.05

**Table 2:** IT foam-model parameters for the flow of 0.5 wt% Amphosol-nitrogen foam in the Bentheimer sandstone, with the original and the modified liquid relative-permeability parameters.

Parameter	*fmmob*	*epdry*	*fmdry*	*fmcap*	*epcap*
Surfactant-free k_rw_	6.173 × 10^5^	11197	0.267	1.503 × 10^−5^	0.609
Modified k_rw_	6.173 × 10^5^	11119	0.199	1.490 × 10^−5^	0.609
Single *k*_*rw*_	2.50 × 10^6^	83335	0.146	8.335 × 10^−5^	2.0
